# Turbidity, Waterfowl Herbivory, and Propagule Banks Shape Submerged Aquatic Vegetation in Ponds

**DOI:** 10.3389/fpls.2018.01514

**Published:** 2018-10-16

**Authors:** Stijn Van Onsem, Ludwig Triest

**Affiliations:** Plant Biology and Nature Management, Department of Biology, Vrije Universiteit Brussel, Brussels, Belgium

**Keywords:** submerged macrophytes, phytoplankton, avifauna, Anatidae, seeds, oospores, biomanipulation

## Abstract

The aquatic vegetation in nutrient-rich shallow lakes and ponds is structured by the interplay of multiple biotic and abiotic drivers. We tested the contribution of the macrophyte propagule bank and the delayed as well as direct impact of waterbirds on submerged aquatic vegetation in a peri-urban pond system. To clarify the functional hierarchy of predictor variables, effects of herbivorous waterfowl and propagule bank potential were ranked relative to environmental, phytoplankton, and zooplankton indicators. Two aspects of the aquatic vegetation – community composition and total pond-scale cover – were discriminated. Within vegetation communities, phytoplankton biovolume and waterfowl herbivory during summer were linked to low macrophyte abundance, whereas propagule density of angiosperms was positively associated with specific assemblages of submerged macrophytes. High algal biovolume and summer waterfowl grazing seemed to affect maximal pond-scale cover of submerged aquatic vegetation. The presence of waterfowl in cold and spring periods was unrelated to vegetation structure in the consecutive main growth season. In addition, availability of propagules in the sediment did not automatically prompt pond-wide vegetation cover (especially when overruled by high waterfowl densities), nor did it guarantee a position in the submerged macrophyte community. Nonetheless, propagule bank potential was related to the waterbody’s general ecological status, since turbid ponds exhibited impoverished propagule reserves compared to ponds residing in a clear, macrophyte-dominated state. Inadequate recruitment therefore represents a plausible bottleneck for macrophyte establishment. We conclude that phytoplankton-caused turbidity and high waterfowl biomass densities greatly restrict submerged macrophyte abundance. Propagule banks also participate in structuring submerged aquatic vegetation, though a stronger role is reserved for herbivorous waterfowl.

## Introduction

The aquatic ecosystem of shallow lakes and ponds is molded by a complex set of interacting biotic and abiotic components (Jeppesen et al., 1997; Søndergaard et al., 2003; Scheffer, 2004). Submerged macrophytes, containing plants and macroalgae adapted to underwater life, play a decisive role in shallow waterbodies by promoting high water clarity (Scheffer et al., 1993) and increased biodiversity in a number of vulnerable faunal groups (De Meester et al., 2006). In nutrient enriched standing waters, submerged aquatic vegetation (SAV) risks collapse when confronted with high phytoplankton-caused turbidity or stress associated with increased productivity of other autotrophic competitors, including periphyton and free-floating plants (Jones and Sayer, 2003; Scheffer et al., 2003; Hidding et al., 2016). The occurrence and persistence of phytoplankton blooms – often the result of high fish densities – can lead to an alternative stable state as opposed to the macrophyte-dominated equilibrium (Scheffer et al., 1993).

Because light availability is such an essential requirement for SAV development, the effect of phytoplankton abundance and zooplankton filtering has been studied extensively (Burns, 1969; Knoechel and Holtby, 1986; Sayer et al., 2010). Nonetheless, other features of the freshwater ecosystem directly or indirectly influencing macrophyte growth have been identified. For one, aquatic vegetation tends to be more sensitive to herbivory compared to terrestrial plants, due to the relatively high palatability of submerged macrophyte species (Lodge, 1991; Bakker et al., 2016). The intensity of waterfowl grazing inversely relates to the standing crop of wetland vegetation, provided that bird counts are converted into biomass density (kg/ha; Wood et al., 2012).

The potential impact of waterfowl has inspired research focusing on SAV dynamics (Irfanullah and Moss, 2004; Hilt, 2006; Gayet et al., 2011; Gyimesi et al., 2011; van Altena et al., 2016). Nevertheless, birds are easily overlooked during routine assessment of macrophyte dynamics because of their mobility and migratory habits, especially outside major wetland areas or in regions not experiencing striking seasonal peaks in waterfowl numbers. As a consequence, studies on avifauna-SAV interactions in pond systems are currently scarce compared to those in large wetlands (Hangelbroek et al., 2002; Hidding et al., 2010a).

A further element plausible to affect the composition of SAV is the macrophytic propagule bank (Bakker et al., 2013), the collection of sexually or vegetatively produced dispersal and survival units accumulated over time in the sediment. In charophycean macroalgae, sexually generated oospores usually form the dominant propagation mode (Bonis and Grillas, 2002). Larger variation exists within angiosperm macrophytes, with the production of seeds, turions, tubers, and rhizomes as well as less-specialized fragments (Grace, 1993; Barrat-Segretain, 1996).

Although the composition of the submerged propagule bank does not necessarily match the standing vegetation (Arthaud et al., 2012), it forms the foundation of new macrophyte emergence after an unfavorable season (Liu et al., 2006) or a major disturbance (Combroux et al., 2001), including water drawdown during biomanipulation (Peretyatko et al., 2012). Therefore, spontaneous recovery of SAV will depend at least partly on the presence of propagules. It remains an open question, however, to what extent a rich, dense propagule bank could guide the ecosystem toward the desired clear, macrophyte-dominated equilibrium under nutrient-rich conditions (Hilt, 2015), and how strongly individual species rely on input from propagules to gain a foothold within the vegetation.

In view of the broad spectrum of drivers affecting cover and assemblage of SAV, it is recommended to simultaneously explore their significance. Acknowledging the relevance of, as well as the interaction among, predictor variables will greatly substantiate and streamline conservation efforts directed at restoring aquatic vegetation (Madsen et al., 2001; Case and Madsen, 2004; Irfanullah and Moss, 2004; Liess and Hillebrand, 2004; Gulati et al., 2008; Hidding et al., 2016).

In this study, we ordered an elaborate set of candidate parameters involved in SAV development in a temperate, nutrient-rich, peri-urban pond system. Data on herbivorous waterfowl and macrophyte propagule banks – two freshwater components rarely credited in smaller waterbodies – were incorporated to test their role in proportion to physical–chemical, nutrient, phytoplankton as well as zooplankton indicators. We hypothesized that (1) phytoplankton abundance would be particularly pertinent, and that (2) waterfowl negatively, and (3) propagule banks positively influence submerged macrophytes. The impact mechanism of bird populations was predicted to vary periodically (4), due to postponed effects triggered by consumption outside of the growth season peak. The occurrence of macrophyte species within the vegetation (5), as well as the ponds’ general ecological status (6), was expected to echo the composition and richness of the propagule bank. Finally, we hypothesized that (7) the relevance of predictor variables is species-specific and, additionally, depends on the distinction between the vegetation community (macrophyte assemblage aspect) and SAV extent at the scale of the waterbody (total macrophyte cover aspect).

## Materials and Methods

### Study Area and Conditions

**FIGURE 1:**
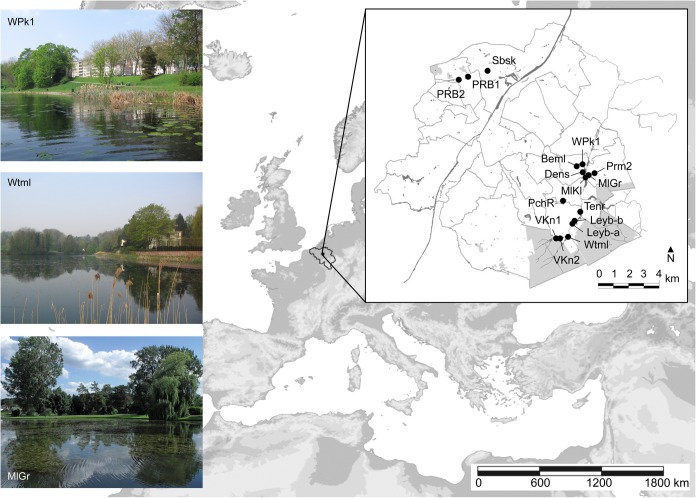
Location of the Brussels-Capital Region (gray area: forest) with 16 studied ponds. Left insets: examples of ponds.

The study was conducted in 2009 and 2010 in 16 shallow man-made ponds located at the outskirts of the Brussels-Capital Region, Belgium (**Figure 1**). Historically, all ponds have been excavated along stream courses, and generally a flow-through hydrological regime is maintained. Thirteen ponds are situated in the Woluwe catchment, a small river largely fed by forested headwaters. Eleven ponds are situated within parks with broad grass lawns, while the remaining five have perimeters that are predominantly forested. Pond area ranges from 0.11 to 3.57 ha (average 1.10 ha), with depths spanning from 0.47 to 1.30 m (average 0.90 m).

As a result of past anthropogenic eutrophication largely sustained through internal loading, nutrient levels in ponds of the Brussels-Capital Region typically correspond to hypereutrophic conditions (OECD, 1982). To battle algal and cyanobacterial blooms and increase ecological value, all ponds had been biomanipulated within the past decade prior to the study. Biomanipulation in the region typically encompasses water drawdown with subsequent fish removal (Peretyatko et al., 2012). The short-term success of this management procedure has proven to be almost complete (De Backer et al., 2012), so that most ponds at least during the initial phase following biomanipulation reside in a clear-water state and develop SAV. In one pond, however (PchR), active biological interference was limited to stocking with juvenile Northern pike (*Esox Lucius* L.), without success. Three ponds (MlGr, MlKl, and Prm2) were emptied early 2009, refilled after the growth season and only sampled in the summer of the next year. At the time of sampling, at least two upstream ponds were presumably fishless (Beml, Dens; De Backer et al., 2012), while in several others large benthivorous fish and/or schools of zooplanktivores were observed (Leyb-a, Leyb-b, PchR, PRB1, PRB2, Prm2, Tenr, VKn2, and WPk1).

### Propagule Bank

#### Sediment Collection

Sediment was collected in spring (April) 2009 with a core sampler (Eijkelkamp multisampler; diameter 40 mm) in three non-overlapping transects per pond. Length, direction and spacing of transects varied in function of pond geometry (size and morphology), and were chosen to maximize representation of the main non-littoral zones of the waterbody. Along each transect and at equal intervals, we stopped at five stations and took three subsamples per stop (15 subsamples in total). Sediment cores were divided into an upper layer of 5 cm (supposed to be the most ecologically active; Dugdale et al., 2001; Bonis and Grillas, 2002; Spencer and Ksander, 2002) and a lower layer from a depth of 5–10 cm (Csontos, 2007) used for comparability and potentially acting as a refuge (Ozimek, 2006). This way, the sediment from five stops per transect was pooled into two samples, representing either the top or the lower sediment layer (abbreviated ‘top’ and ‘sub’). In total, 90 samples were collected in 16 ponds. The sediment was stored for 2.5 months at 4°C, to extend the cold stratification period and increase the likelihood of germination (Hay et al., 2008).

#### Germination Experiment

The seedling-emergence method described by Ter Heerdt et al. (1996) was adopted to germinate viable macrophyte propagules in a greenhouse. Sediment samples were gently homogenized. Fine mineral and organic particles of a known volume (generally 0.7 L) were removed using a sieve tower with smallest mesh size of 0.212 mm, suited to retain most charophycean oospores (Haas, 1994). The remaining fractions were pooled and spread out as a thin layer in plastic trays (surface area: 34 × 21 cm, height: 8 cm) filled with 1 cm of sand on top of a 1.5-cm-thick nourishing layer (mixture of sand and potting soil in a 4:1 proportion). All substrate material had been sterilized prior to the germination trials. Trays containing the samples were perforated and kept in larger, outer trays, to facilitate addition of water without sediment disturbance.

Samples were immediately submerged in a mix of equal proportions distilled and tap water. The water level was maintained at ca. 5 cm during the growth experiment. We chose to inundate our samples instead of keeping them waterlogged, to avoid excessive germination of emergent taxa and to increase germination success in submerged species (Boedeltje et al., 2002; Xiao et al., 2010). *Daphnia* were allowed to emerge from ephippia and redistributed over the samples if necessary. The next 6 months, macrophyte seedlings were picked from the sediment, identified and counted on regular basis. Counts of non-identifiable *Chara* germlings were redistributed based on relative proportions of confirmed identifications within the sample.

At the end of the experiment, the sample layer was recovered and scanned for ungerminated, non-oospore propagules (mainly angiosperm seeds). As a number of thus retrieved fruits (mainly achenes belonging to *Potamogeton* spp.) still spontaneously germinated afterward, all seeds were considered viable but dormant and added to counts obtained from the germination trials. Final propagule densities were converted to numbers per liter sediment (equivalent to 1/50th of a m^2^). Shannon indices (H’) were calculated as a measure of diversity within the top and sub layers.

In statistical analyses, angiosperm and charophycean propagule densities were considered separately, since reproductive strategies of submerged species of Angiospermae generally involve investing in fewer, heavier propagules compared to Charophyceae (Haas, 1994; Bonis and Grillas, 2002; Kleyer et al., 2008).

### Vegetation Survey

We evaluated two ecological aspects of the aquatic vegetation: (1) the community composition, and (2) total pond-scale cover of SAV. Firstly, macrophytes growing along the three sediment sampling transects (including five stops/transect) were monitored twice, once in July 2009 and again in July 2010. From a boat, a floating PVC frame (1 m^2^) was placed on top of the macrophyte assemblage followed by species cover estimation. Low-growing vegetation, as well as the floor of turbid ponds, was sampled using a rake. Vertical stratification of vegetation was taken into account, allowing the combined cover to exceed 100%.

Secondly, in both years waterbody-scale relative cover of submerged macrophytes was visually surveyed by traversing the pond in several directions, on three occasions: end of May, beginning of July and mid August. The maximal SAV extent during the growth season was used in the statistical analyses.

### Environmental Variables, Phytoplankton, and Zooplankton

During two campaigns per year (end of May and at the time of vegetation community analysis in July), we measured a number of environmental variables (**Table 1**): water depth, Secchi depth, physical–chemical variables (temperature, pH, conductivity, and oxygen concentration) and nutrients (NO_x_, sum of nitrate and nitrite; NH_4_^+^, ammonium; SRP, soluble reactive phosphorus; TP, total phosphorus). When the Secchi disk was still detectable at the bottom, a correction was added in function of disk visibility (either 0.5, 0.3, or 0.1 m). Per pond, a water volume of 10 L was collected with a vertical tube sampler for measurements of physical–chemical variables (recorded on the field with a portable WTW Multi 340i multimeter) and nutrient analysis. Nutrient subsamples were frozen upon return and concentrations were analyzed using spectrophotometry (APHA, 1995).

**Table 1:** Variables considered in multivariate community analysis and multiple regression of pond-scale SAV cover predictors.

Type	Variable abbreviation	Variable full	Unit	Proxy^a^	SAV community (RDA)	SAV cover (multiple regression)
Abiotic						
Physical–chemical	Cond	Conductivity	μS/cm	Cond	X^∗^	X
	O_2_	Oxygen concentration	mg O_2_/L	pH		
	pH	pH		pH	X^∗^	X^∗^
Nutrients	DIN	Dissolved inorganic nitrogen	mg N/L	DIN	X	X
	NH_4_^+^	Ammonium	mg N/L	DIN		
	NO_x_	Nitrate + nitrite	mg N/L	DIN		
	SRP	Soluble reactive phosphorus	mg P/L	TP		
	TP	Total phosphorus	mg P/L	TP	X	X
Physical	D	Depth	m	D	X	X
	SD	Secchi depth	m	SD/D		
	SD/D	Secchi depth/depth		SD/D	X	X
Pond characteristics	TSB	Time since biomanipulation	years	TSB	X	X
Biotic						
Phytoplankton	Chl *a* + phaeo	Chl *a* + phaeopigments	μg/L	Biovol		
	Biovol	Biovolume	mm^3^/L	Biovol	X^∗^	X^∗^
Zooplankton	LCL	Large Cladocera length	mm	LCFR		
	LCD	Large Cladocera density	n/L	LCFR		
	LCFR	Large Cladocera filtering rate	mL/L/day	LCFR	X	X
Propagule bank	T_Charo	Top layer Charophyceae prop. density	n/L	T_Charo	X	X
	S_Charo	Sub layer Charophyceae prop. density	n/L	T_Charo		
	T_Angio	Top layer Angiospermae prop. density	n/L	T_Angio	X^∗^	X
	S_Angio	Sub layer Angiospermae prop. density	n/L	T_Angio		
	T_Shannon	Top layer Shannon H’	H’	T_Angio		
	S_Shannon	Sub layer Shannon H’	H’	T_Angio		
Macrophytes	MF_FM	Cover free-floating + floating-leaved macrophytes	%	MF_FM		X
Waterfowl	WF_Cold	Waterfowl cold period biomass density	kg/ha	WF_Cold	X	X
	WF_Spring	Waterfowl spring period biomass density	kg/ha	WF_Spring	X	X
	WF_Warm	Waterfowl warm period biomass density	kg/ha	WF_Warm	X^∗^	X^∗^

*^a^Proxy: strongly correlated (r_s_> 0.7) variable selected for multivariate and regression analyses. Original list has been reduced according to covariate structure, retaining key variables of interest (X). Asterisk, significant or relevant.*

Additionally, we integrated results of phytoplankton and zooplankton analyses cumulated in an internal database (**Table 1**). Phytoplankton and zooplankton samples had been collected end of May and beginning of July using a tube sampler and a water volume of 10 L. Two measures of phytoplankton abundance were extracted: pigment concentrations (Chl *a* + phaeophytin) and biovolume.

For zooplankton, we used identifications and length measurements of large Cladocera (mainly *Daphnia*; functional group as defined by Moss et al., 2003) to calculate both density (LCD, Large Cladocera Density) and mean length (LCL, Large Cladocera Length) per pond and growth season. Both measures of zooplankton grazing efficiency have been tested in Brussels peri-urban ponds, with LCL generally displaying the strongest relation with pond turbidity level (Peretyatko et al., 2009; De Backer et al., 2014). Also, LCL can be considered to be a surrogate for zooplanktivorous fish predation, because of down-regulation of cladoceran size (Brooks and Dodson, 1965).

To further improve potential predictive power of zooplankton on SAV structure, we combined information on density and body size in a metric predicting the filtering rate of the total population of large Cladocera (LCFR, Large Cladocera Filtering Rate; Reynolds, 2006). Calculating individual filtering rate for 20 specimens of known size (incorporating temperature data; Burns, 1969) and multiplying this rate with LCD provided an indication of the grazing potential, expressed as the total volume of water filtered per day by large zooplankters present in 1 L of pond water (Reynolds, 2006).

For environmental, phytoplankton, and zooplankton variables, the warm-period average (May and July) for each pond was used in statistical analyses.

### Waterfowl

Effect of fully or partially herbivorous avifauna species on macrophytes was tested using records obtained from a regional database compiled with year-round input from various sources, including citizen science (Leefmilieu Brussel^1^). These data are reliable since a large fraction is validated by experts on the basis of attached images or originates from experienced bird-watchers. Coordinates were used to assign bird observations to particular ponds. A 50 m buffer extended the zone of interest to surrounding green areas potentially containing resting, roosting or grazing waterfowl. If a clear obstacle was present between the observation and a particular pond, or if distance to a nearby pond was shorter, the count was not retained. Only waterfowl species that potentially include macrophytes in their diet were selected (Wood et al., 2012).

Since the type and magnitude of waterfowl herbivory differs between macrophyte growth stages, avifauna observations were partitioned over three time frames per year, roughly corresponding to meteorological seasons and the pattern of bird migration. This allowed separating effects of belowground herbivory in autumn and winter from primarily direct grazing in the sprouting phase and the main growth period. During vegetation senescence and throughout the major part of autumn and winter, conjointly termed the *cold* period (WF_Cold; September 1st – February 28th), herbivory would mainly impact the macrophyte propagule bank (Perrow et al., 1997; Hidding et al., 2010b). On the other hand, the *spring* period or recruitment phase (WF_Spring; March 1st – May 15th) is known to be a delicate window of opportunity for macrophyte establishment (Marklund et al., 2002). Finally, during the *warm* period following initial SAV expansion (WF_Warm; May 16th – August 31st, including the peak of summer), herbivory likely affects both standing crop biomass and propagule production (Hidding et al., 2010b; Gayet et al., 2011; Marco-Méndez et al., 2015). In total, bird observations spanned September 1st 2008 till August 31st 2010.

Bird counts were converted to a measure of effective *herbivorous* biomass, by multiplying them with species-specific average biomass and percentage plant material (including seeds) in diet, provided by Wood et al. (2012). Because of supposedly substantial amounts of multiplicates and false negatives in the observation database, we used the maximum herbivorous biomass per period and pond as a proxy for the influence of a species in that particular period, and summed the species maxima to obtain the total herbivorous biomass of the avifauna community. Finally, total herbivorous biomass was divided by pond size. The resulting biomass density (kg/ha) represents a potential peak in waterfowl-induced stress (Wood et al., 2012). Features of the aquatic vegetation in July were related to waterfowl herbivorous biomass density in the cold and spring periods immediately prior to, and the warm period during, the summer of macrophyte survey.

### Data Analysis

#### Propagule Bank, Waterfowl, and SAV Species

Species-specific patterns in abundance of submerged macrophyte species in relation to propagule density and waterfowl pressure were graphically explored by means of descriptive statistics. The potential of the propagule bank and of SAV in predicting species occurrence in vegetation of subsequent summer season(s) is presented using the overlap in species incidence between physical compartments (propagules in sediment versus standing vegetation).

#### Propagule Bank and Pond Ecological Status

In order to determine the relationship of the propagule bank with the general condition of the ecosystem, ponds were categorized in function of the pond-scale SAV cover and their turbidity level. Thresholds were 30% maximal pond-scale SAV cover (marking the lower boundary of ecosystems stabilized by submerged macrophytes; Jeppesen et al., 1990; De Backer et al., 2012) and 20 mm^3^/L averaged phytoplankton biovolume (indicating a high turbidity; Peretyatko et al., 2007). Three classes were created, representing different levels of ecological status: clear-water, highly vegetated (macrophyte-dominated) ponds (*Clear H*; ≥30% SAV cover and <20 mm^3^/L phytoplankton biovolume; *n* = 17 over the study period), clear-water ponds with low vegetation cover (*Clear L*; <30% SAV and <20 mm^3^/L biovolume; *n* = 7) and turbid ponds without aquatic vegetation (*Turbid*; <30% SAV and ≥20 mm^3^/L biovolume; *n* = 5).

The relationship of propagule densities with the pond ecological state was tested using zero-inflated generalized linear mixed models (ZIGLMM; Bolker et al., 2009; Zuur et al., 2009) with pond status as a fixed factor and pond-year nested within status as a random factor. Pond in a given year was treated as blocking factor since ponds contained three transects and are reset during winter, which allowed them to switch status between years. Within the framework of the study, the propagule bank was considered to be a relatively invariable element of pond identity. Therefore, results from the germination experiment performed in the first year were coupled to pond status as observed in the first as well as the second year. This assumption is backed by the high similarity between top and sub layers in terms of propagule contents (see the Section “Results”), an indication of propagule bank continuity at short temporal scale.

Patterns in angiosperm and charophycean propagule densities as well as top and sub sediment layers were separately analyzed. Furthermore, we calculated Chao-Sørensen abundance-based similarity (Chao et al., 2005, 2006) among propagule bank and developed vegetation. The Chao-Sørensen index ranges from 0 (no shared species) to 1 (complete overlap) and integrates information on species incidence and their relative abundance. We excluded emergent taxa, included free-floating and floating-leaved species and ignored the non-vegetated portion of survey plots. If both propagule bank and the corresponding vegetation transect lacked macrophytes, similarity values were fixed at 1.

ZIGLMM analysis was performed with the *glmmTMB* package in R3.2.0 (Magnusson et al., 2017). Models for propagule densities were fitted using the negative binomial distribution, because of overdispersion in the count data (*k* < 10; Bolker, 2007). Beta regression was selected for Chao-Sørensen similarities (extremes 0 and 1 were slightly adjusted for this purpose). The significance threshold was set at 0.05.

#### Predictors of Macrophyte Community

For multivariate analysis, transect data were averaged per pond. Prior to ordination, macrophyte cover was Hellinger transformed (Legendre and Gallagher, 2001). The non-vegetated portion (i.e., bare sediment) within a quadrat was entered as an additional species variable. As a direct gradient analysis, we used Redundancy Analysis (RDA). The original list of environmental variables was a mix of biotic (*n* = 14) and abiotic (*n* = 12) variables potentially interacting with macrophyte growth (**Table 1**). Additionally, the time since last biomanipulation (TSB, number of years) was included, given the tendency for gradual deterioration following successful fish removal (Søndergaard et al., 2008; see **Supplementary Material**).

Explanatory variables were log-transformed. Spearman’s rank-order correlations were used to eliminate multicollinearity: one key variable was selected for each group of strongly related parameters (*p* < 0.05 and *r*_s_> 0.7; **Table 1**). The resulting 14 abiotic and biotic variables avoided multicollinearity and were used in the final RDA. Significance was tested using forward selection, with Monte Carlo permutations (999 repetitions) under restricted model. A PCA with supplementary variables was used to illustrate the relationship between macrophyte species and the environment. Ordination was performed in CANOCO 4.5 (ter Braak and Šmilauer, 2002).

#### Predictors of SAV Pond-Scale Cover

The maximal pond-scale cover of submerged vegetation in summer (May–August) was used as a response variable in multiple regression. Predictor variables in this analysis were taken from the reduced set of variables given in **Table 1**. A further reduction was achieved using the output from simple regressions between dependent and single independent variables. All models were fitted using beta distributions (*betareg* package; Cribari-Neto and Zeileis, 2010), because of the proportional nature of the data (ranging from 0, SAV absent, to 1, complete cover). A small correction was applied to all 0 and 1 values.

After acquiring the shortlist of potentially relevant variables, multimodel inference (*AICcmodavg* package; Mazerolle, 2016) was applied on all possible models, to estimate the contribution of these variables in predicting SAV cover (Burnham and Anderson, 2002; Grueber et al., 2011). An AICc-based (Akaike Information Criterion for finite sample size) threshold to retain only top-ranked models was not deemed necessary because of the relatively small set of predictors. The explanatory power of predictors was evaluated using the weighted estimates and their 95% confidence intervals (CI; Grueber et al., 2011).

## Results

### Abiotic Variables, Phytoplankton, and Zooplankton

Summer values of abiotic and biotic variables are given in **Supplementary Material**. Conductivity was high (>800 μS/cm) in Beml, PRB1, Prm2, and WPk1, and low (<500 μS/cm) in ponds supplied by forest streams (VKn1, VKn2, Wtml). A few ponds experienced hypoxic conditions, due to high lemnid or floating-leaved plant cover. Most ponds were characterized by low acidity values (average pH of 8.1 ± 0.5 SD). DIN varied widely, with an average of 0.226 ± 0.294 SD mg N/L. Total phosphorus status of the ponds also fluctuated strongly, but was overall high (average of 0.370 ± 0.374 SD mg P/L). Mean water depth was 0.9 ± 0.2 SD m, while Secchi depth was 1.2 ± 0.5 SD m on average – suggesting light penetration generally permitted SAV colonization.

Transparency levels ranged from very clear (minimum phytoplankton pigment concentration and biovolume were 3.1 μg/L and 0.2 mm^3^/L, respectively) to extremely turbid in ponds experiencing phytoplankton blooms (maximum values were 257.0 μg/L Chl *a* + phaeophytin and 36.6 mm^3^/L biovolume).

In zooplankton communities, *Daphnia* was the dominant large cladoceran genus. A number of ponds contained marginal numbers of *Eurycercus* and *Simocephalus*. Individuals were very small in some ponds (minimum LCL of 0.20 mm in Leyb-b in 2010), or completely absent (LCD = 0 n/L in Leyb-a in 2010). On multiple occasions (especially in fishless ponds), LCL and/or LCD were high, resulting in potential clearance of the complete basins’ volume each day (LCFR > 1 L). LCFR exceeded 3,800 mL/L/day in Sbsk in 2010. Generally, zooplankton parameters varied widely, indicating a broad range of potential filtering capacities (see **Supplementary Material**). LCFR was very low in turbid ponds.

### Waterfowl

In total, 515 waterfowl entries were extracted from the dataset (around 6,300 individuals belonging to 19 herbivorous or omnivorous taxa; see **Supplementary Material**). These included 16 species of Anatidae (ducks, geese, swans, sheldgeese, and Egyptian goose) as well as three Rallidae species (rails). Five common waterfowl species dominated the avifauna in terms of computed total biomass, two of which are non-native to Belgium. In decreasing order of importance, these were *Cygnus olor* Gmelin (Mute swan; 34% of herbivory-related biomass), *Branta canadensis* L. (Canada goose, non-native; 21%), *Alopochen aegyptiacus* L. (Egyptian goose, non-native; 16%), *Anas platyrhynchos* L. (Mallard; 14%) and *Fulica atra* L. (Eurasian coot; 9%).

**FIGURE 2:**
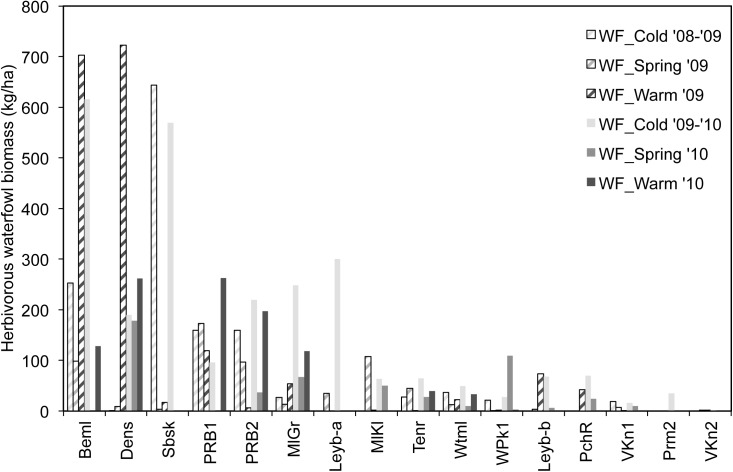
Herbivory-related biomass density of waterfowl in the studied ponds during six seasons. WF_Cold, Waterfowl observed in cold periods; WF_Spring, Waterfowl observed in spring periods; WF_Warm, Waterfowl observed in warm periods. Ponds are arranged according to decreasing average waterfowl density.

The presence of waterfowl was unevenly distributed, both spatially and temporarily (**Figure 2**). Cold period peaks (WF_Cold) matched with Beml and Sbsk (616 and 644 kg/ha), while spring values (WF_Spring) were generally low. Peaks observed during the warm season (WF_Warm) were even more prominent, mainly in Beml and Dens (703 and 723 kg/ha, respectively). Within these ponds’ perimeter, extensive flocks of larger Anatidae species aggregated in summer. With a surface area of 0.43 ha, Beml endured the company of 60 *A. aegyptiacus* and 17 *C. olor* in the warm season of 2009. In Dens (surface area 0.33 ha), 70 *B. canadensis* and three *Chloephaga picta* Gmelin (Magellan goose, non-native) were noticed simultaneously. Average biomass densities for the six consecutive periods were 84 ± 16 SD, 37 ± 52 SD, 110 ± 237 SD, 164 ± 189 SD, 32 ± 49 SD, and 65 ± 97 SD kg/ha.

### Propagule Bank

A total of 11,015 propagules from eight submerged macrophyte species germinated during the study period. Most (95.07%) belonged to three species of Charophyceae: oospores of *Chara vulgaris* L., *C. globularis* Thuillier and *Nitella mucronata* (Braun) Miquel, respectively, accounted for 65.60, 23.09, and 6.38% of all propagules. Angiosperm submerged macrophytes were represented mainly by *Zannichellia palustris* L. (3.07%) and *Potamogeton pusillus* L. (1.71%), accompanied by *P. pectinatus* L. (0.11%), *Callitriche obtusangula* Le Gall ex Hegelm. (0.04%) and *P. crispus* L. (0.01%).

Large differences in presence and densities of submerged species were observed between ponds. Top and sub layers contained on average 290.5 ± 388.2 SD and 107.7 ± 153.2 SD oospores/L, respectively. Mean angiosperm propagule densities were 15.8 ± 23.4 SD n/L in the top sediment layer and 7.9 ± 12.9 SD n/L in the underlying layer. Top and sub layers were correlated in terms of density (Charophyceae: *r*_s_ = 0.86, *p* < 0.0001; Angiospermae: *r*_s_ = 0.90, *p* < 0.0001) and on average showed relatively high Chao-Sørensen similarity (0.66 ± 0.45 SD). Overall, Shannon diversity within the propagule pool of both sediment layers was low, as a result of charophycean dominance and low species richness (H’ top: 0.31 ± 0.35 SD; H’ sub: 0.35 ± 0.36 SD).

A number of ponds were characterized by relatively diverse and/or dense submerged macrophyte propagule banks (Dens, Leyb-a, Leyb-b, MlGr, VKn1 and Wtml, for instance); others displayed poor recruitment potential. In one turbid pond, no propagules sprouted from the collected sediment (PchR). Five ponds lacked angiosperm propagules (PchR, PRB1, PRB2, Prm2, and VKn2), while germinating oospores were entirely absent from only one site (PchR).

### Standing Vegetation

**FIGURE 3:**
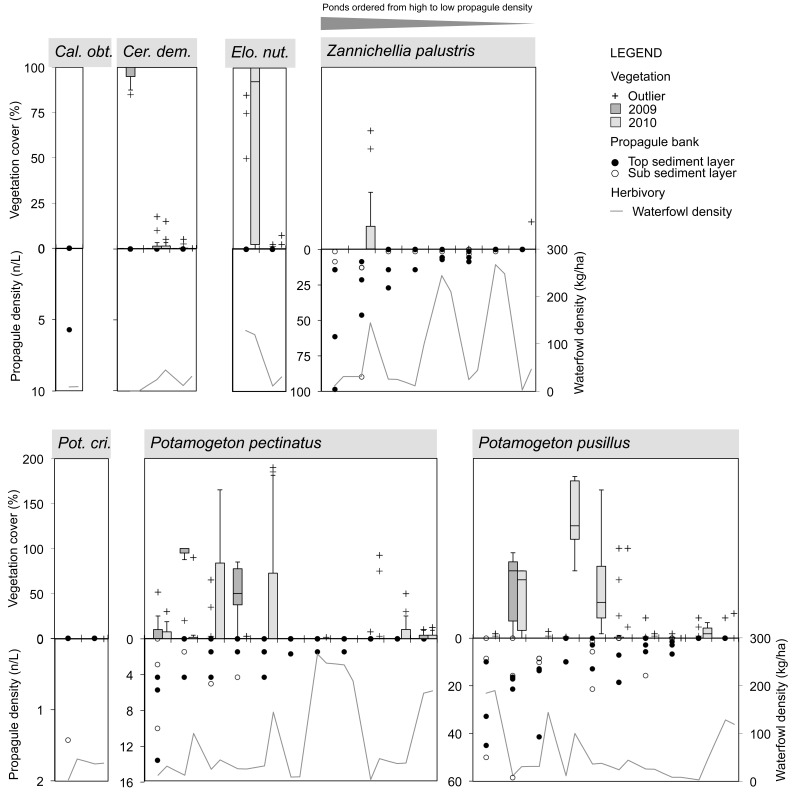
Species-specific relationship for Angiospermae between spring sediment propagule density (lower inverted *Y*-axis on left side; data points separately shown: *n* = 3 for top and sub layer each), herbivorous waterfowl biomass (lower *Y*-axis on right side; average of cold, spring, and warm periods) and established species cover in two consecutive summer seasons (upper *Y*-axis; box = 25th percentile-median-75th percentile, whiskers = non-outlier range, crosses = outliers and extremes, *n* = 15). Ponds (*X*-axis) are ordered from high to low propagule densities, excluding those in which the macrophyte species is absent. *Cal. obt.*, *Callitriche obtusangula*; *Cer. dem.*, *Ceratophyllum demersum*; *Elo. nut.*, *Elodea nuttallii*; *Pot. cri.*, *Potamogeton crispus*.

**FIGURE 4:**
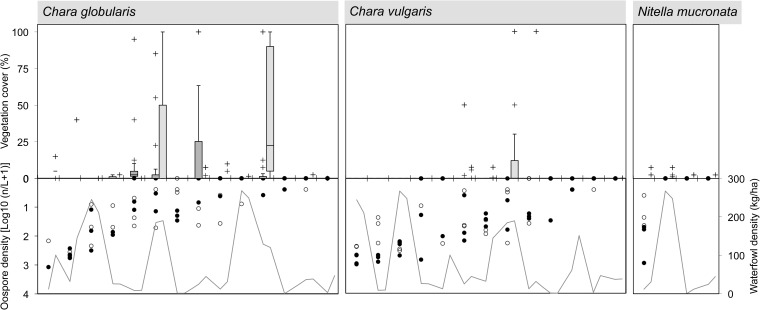
Species-specific relationship for Charophyceae between spring sediment propagule density (lower inverted *Y*-axis on left side; data points separately shown: *n* = 3 for top and sub layer each), herbivorous waterfowl biomass (lower *Y*-axis on right side; average of cold, spring, and warm periods) and established species cover in two consecutive summer seasons (upper *Y*-axis; box = 25th percentile-median-75th percentile, whiskers = non-outlier range, crosses = outliers and extremes, *n* = 15). Ponds (*X*-axis) are ordered from high to low propagule densities, excluding those in which the macrophyte species is absent. For legend, see **Figure 3**. Note logarithmic scale for oospore densities.

**FIGURE 5:**
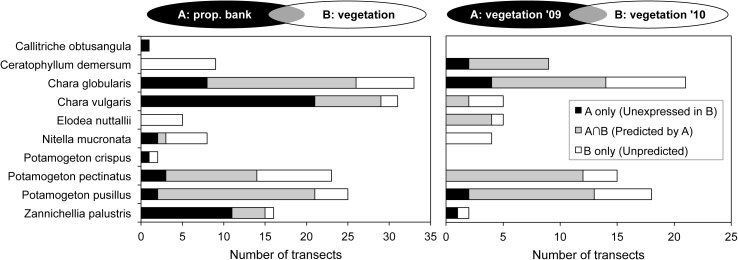
Occurrence of submerged macrophytes and similarity between compartments (A vs. B). Gray areas visualize the predictive potential in terms of overlapping species presence. Left: occurrence as propagules in spring propagule bank (A) and as expressed vegetation in summer (B); right: occurrence within vegetation of 2009 (A) and of the following year (B), excluding ponds that were empty in the first year.

The submerged macrophyte community in July was composed of nine species in total (**Figures 3–5**). A number of ponds completely lacked SAV during one (Beml and Dens in 2009) or both monitoring periods (PchR and PRB2). Others were sparsely colonized with submerged species (≤5% cover; Beml, Dens, Prm2 and VKn1, all in 2010). At macrophyte-rich sites, *P. pectinatus*, *P. pusillus* and *C. globularis* were particularly common, often (co-)dominating the aquatic vegetation. Other submerged species sporadically reaching high cover levels were *Ceratophyllum demersum* L., *C. vulgaris*, *Elodea nuttallii* (Planch.) H. St. John and *Z. palustris*. VKn2 was completely filled with *C. demersum* throughout the study period. Submerged species richness was relatively high in MlGr (five species in 2010), MlKl (five species in 2010), Tenr (six species in both years), and Wtml (four species in 2009 and seven in 2010).

Floating-leaved macrophytes [*Nuphar lutea* (L.) Sm. and *Nymphaea alba* L.] were locally abundant in Beml, WPk1, and Wtml, along the fringes or in smaller patches. During summer, two ponds (Sbsk and VKn1) became gradually covered by lemnids [including *Lemna trisulca* L., *Lemna minor* L., and *Spirodela polyrhiza* (L.) Schleid.], while WPk1 developed extensive detached algal mats [composed of filamentous algae and *Enteromorpha intestinalis* (L.) Nees]. Finally, *Riccia fluitans* L., *Sagittaria sagittifolia* L., and *Veronica anagallis-aquatica* L. were recorded within vegetation transects of a single pond each.

### Macrophyte Species Occurrence in Relation to Propagule Bank and Waterfowl

Macrophyte species exhibited variable relationships with propagule densities and herbivory by avifauna (average of spring, warm, and cold periods), although in this context the influence of both predictor variables on summer vegetation abundance seemed rather weak (**Figures 3**, **4**). Maximum species-specific macrophyte cover values concurred with intermediate propagule densities for *C. globularis*, *C. vulgaris*, *P. pectinatus*, *P. pusillus*, and *Z. palustris*. Nevertheless, for three common angiosperm species (*P. pectinatus*, *P. pusillus*, and *Z. palustris*), maximum cover values were attained at sites with relatively rich propagule banks compared to the pattern observed for charophycean algae. Additionally, *C. demersum*, *E. nuttallii*, *P. pectinatus*, and *P. pusillus* were also able to develop medium or high summer cover along some vegetation transects despite absence of propagules in sediment. *C. obtusangula* was only found within the propagule bank.

For common macrophytes, maximal waterfowl densities – as retrieved for ponds Beml and Dens,– were systematically linked with (near) vegetation absence in summer (**Figures 3**, **4**). In other ponds, the relationship between potential waterfowl herbivory and macrophyte occurrence revealed a fairly chaotic pattern. Some ponds developed high macrophyte cover unhindered by abundant waterfowl residing in or traveling through the area, while in others, species were unable to establish even at low bird biomass densities.

In terms of species incidence, the predictive power of propagule bank and of vegetation in the first year differed among macrophytes (**Figure 5**). *C. obtusangula*, *C. vulgaris*, and *Z. palustris* showed comprehensive unrealized potential, since few viable seeds and oospores managed a transition toward standing populations (**Figure 5**, left). Other species (*C. globularis*, *P. pectinatus*, and *P. pusillus*) expressed their recruiting abilities in the majority of transects. On the other hand, for all species presence within the macrophyte community was not exclusively explained by propagule availability in spring.

Within the vegetation, continuity over consecutive years was high for *C. demersum*, *E. nuttallii* and two common *Potamogeton* species (especially *P. pusillus*), but less for charophycean macroalgae and *Z. palustris* (**Figure 5**, right).

### Relationship Between Propagule Bank and Pond Ecological Status

In 2009, two ponds were turbid (Turbid), three were clear but lacked abundant SAV (Clear L), while eight resided in a macrophyte-dominated state (Clear H; see **Supplementary Material**). In addition, in the first year three newly biomanipulated ponds were completely drawn down for the duration of the growth season. The ensuing year, the three re-inundated ponds showed high water transparency, yet only two were extensively colonized by submerged macrophytes (MlGr and MlKl). In 2010, three ponds were turbid, four had an intermediate ecological state (Clear L) and nine were abundantly vegetated (Clear H). In between years, three ponds switched categories: Leyb-b (from Clear H to Turbid), VKn1 (from Clear H to Clear L) and WPk1 (from Clear L to Clear H).

**FIGURE 6:**
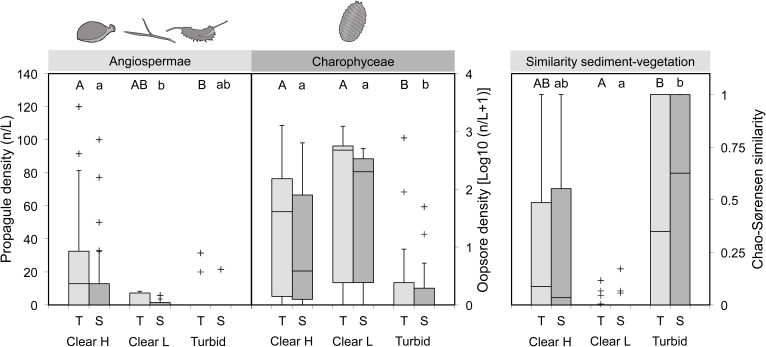
Differences in propagule density (left: Angiospermae – turions, tubers, seeds; middle: Charophyceae – oospores) and abundance-based similarity (right) between ponds grouped according to ecological status (Clear H: clear ponds with high SAV cover; Clear L: clear ponds with low SAV cover; Turbid: turbid ponds devoid of SAV). A distinction is made between top (T) and sub (S) sediment layers. box = 25th percentile-median-75th percentile, whiskers = non-outlier range, crosses = outliers and extremes. Significant differences within a specific sediment layer (ZIGLMM output) are marked with contrasting letters (T: capital letters; S: lower case).

Compared to macrophyte-dominated waterbodies, turbid ponds featured significantly lower propagule densities in the ecologically accessible top sediment layer (Angiospermae: *p* = 0.015; Charophyceae: *p* = 0.022; **Figure 6**, left and middle; **Table 2**). In clear, vegetation-poor ponds the same layer was characterized by intermediate angiosperm propagule densities, but significantly richer oospore reservoirs in comparison to turbid ponds (*p* = 0.031). In the deeper layer, the situation was similar for Charophyceae (Clear H vs. Turbid: *p* = 0.018; Clear L vs. Turbid: *p* = 0.007), while for Angiospermae there was a significant difference between Clear H and Clear L ponds (*p* < 0.001), with Turbid ponds positioned in-between. Chao-Sørensen similarity between top or sub layer contents and vegetation was highly variable in Clear H and Turbid ponds, although the former group had a tendency for low overlap between compartments (**Figure 6**, right and **Table 2**). Similarity was extremely low in Clear L ponds and significantly weaker compared to Turbid ponds.

**Table 2:** Properties of propagule banks and similarity with vegetation in ponds with different ecological status (Clear H, clear ponds with high SAV cover; Clear L, clear ponds with low SAV cover; Turbid, turbid ponds).

Layer	DV Reference category	Descriptives			Contrasted category								
					Clear H			Clear L			Turbid		
		Min	Median	Max	Est.	*SE*	*p*	Est.	*SE*	*p*	Est.	*SE*	*p*
**Top**	*Angiospermae*												
	Clear H	0.0	12.9	120.0	–	–	–	-2.01	1.44	0.164	-4.70	1.93	0.015^*^
	Clear L	0.0	0.0	8.3	2.01	1.44	0.164	–	–	–	-2.70	2.15	0.209
	Turbid	0.0	0.0	31.4	4.70	1.93	0.015^*^	2.70	2.15	0.209	–	–	-
	*Charophyceae (log_10_)*												
	Clear H	0.0	1.6	3.1	–	–	–	0.28	1.54	0.854	-4.32	1.89	0.022^*^
	Clear L	0.0	2.7	3.1	-0.28	1.54	0.854	–	–	–	-4.61	2.13	0.031^*^
	Turbid	0.0	0.0	2.9	4.32	1.89	0.022^*^	4.61	2.13	0.031^*^	–	–	-
	*Chao-Sørensen similarity*											
	Clear H	0.00	0.09	1.00	–	–	–	-1.19	0.61	0.051	1.07	0.67	0.112
	Clear L	0.00	0.00	0.12	1.19	0.61	0.051	–	–	–	2.26	0.80	0.005^**^
	Turbid	0.00	0.35	1.00	-1.07	0.67	0.112	-2.26	0.80	0.005^**^	–	–	-
**Sub**	*Angiospermae*												
	Clear H	0.0	0.0	100.0	–	–	–	-3.04	0.65	2.9E-6^∗∗∗^	-0.24	1.37	0.861
	Clear L	0.0	0.0	5.7	3.04	0.65	2.9E-6^∗∗∗^	–	–	–	2.80	1.46	0.056
	Turbid	0.0	0.0	21.4	0.24	1.37	0.861	-2.80	1.46	0.056	–	–	-
	*Charophyceae (log_10_)*												
	Clear H	0.0	0.6	2.8	–	–	–	1.17	1.35	0.387	-4.04	1.71	0.018^*^
	Clear L	0.0	2.3	2.7	-1.17	1.35	0.387	–	–	–	-5.21	1.91	0.007^**^
	Turbid	0.0	0.0	1.7	4.04	1.71	0.018^*^	5.21	1.91	0.007^**^	–	–	-
	*Chao-Sørensen similarity*											
	Clear H	0.00	0.03	1.00	–	–	–	-0.91	0.51	0.073	1.05	0.56	0.061
	Clear L	0.00	0.00	0.17	0.91	0.51	0.073	–	–	–	1.96	0.67	0.004^**^
	Turbid	0.00	0.63	1.00	-1.05	0.56	0.061	-1.96	0.67	0.004^**^	–	–	-

*Significance codes ^∗^p < 0.05; ^∗∗^p < 0.01; ^∗∗∗^p < 0.001. ZIGLMM results are shown under ‘Contrasted category,’ as crossed with the reference category. DV, dependent variable; Est., estimate; SE, standard error; p, significance value. Significant differences are indicated with an asterisk.*

### Predictors of Macrophyte Community

**FIGURE 7:**
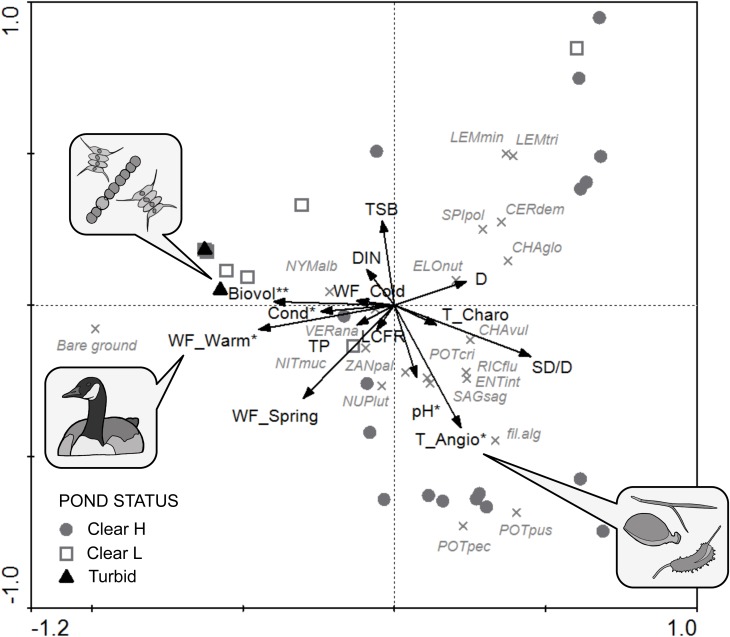
PCA triplot of aquatic vegetation communities including supplementary variables (arrows), species (crosses) and sites (symbols). Variables with a significant conditional contribution after RDA forward selection are marked with an asterisk (^∗^*p* < 0.05; ^∗∗^*p* < 0.01). See **Table 1** for codes of environmental variables. Species abbreviations: CERdem, *Ceratophyllum demersum*; CHAglo, *Chara globularis*; CHAvul, *C. vulgaris*; ELOnut, *Elodea nuttallii*; ENTint, *Enteromorpha intestinalis*; fil. alg, filamentous algae; LEMmin, *Lemna minor*; LEMtri, *L. trisulca*; NITmuc, *Nitella mucronata*; NUPlut, *Nuphar lutea*; NYMalb, *Nymphaea alba*; POTcri, *Potamogeton crispus*; POTpec, *P. pectinatus*; POTpus, *P. pusillus*; RICflu, *Riccia fluitans*; SAGsag, *Sagittaria sagittifolia*; SPIpol, *Spirodela polyrhiza*; VERana, *Veronica anagallis-aquatica*; ZANpal, *Zannichellia palustris*.

The first two axes of the RDA explained 37.8% of variance in the species data and 58.1% of variance in the species-environment relationship (see **Supplementary Material**). In the PCA (**Figure 7**), Clear H ponds were directed toward the clear end of the spectrum (corresponding to higher SD/D values) and clustered in two subgroups, the first being species-rich and dominated by *P. pectinatus* and *P. pusillus* (associated with higher values of T_Angio and pH), the second related to high *C. globularis*, *C. demersum* and lemnid cover. With a few exceptions, Clear L sites grouped together with Turbid ponds, sharing high values of bare ground. Typical vegetation attributes (i.e., low abundance of macrophytes) of these two ecological categories were linked with high phytoplankton biovolume (Biovol), conductivity (Cond), and waterfowl herbivory in the warm period (WF_Warm).

Forward selection retained five significant contributors to macrophyte community composition (conditional effects; **Table 3**), with decreasing effect size: phytoplankton biovolume (Biovol; λ = 0.10; *p* = 0.004), angiosperm top-layer propagule density (T_Angio; λ = 0.09; *p* = 0.019), waterfowl herbivorous biomass density in the warm period (WF_Warm; λ = 0.08; *p* = 0.024), pH (λ = 0.07; *p* = 0.031) and conductivity (Cond; λ = 0.06; *p* = 0.026). Nutrients and cladoceran filtering rate (LCFR) behaved neutral with respect to macrophyte assemblages.

**Table 3:** RDA results for marginal (unique) and conditional (within the constructed model) effects of environmental variables measured in ponds.

Marginal effects		Conditional effects		
Variable	Lambda1	Variable	LambdaA	P
T_Angio	0.09	Biovol	0.10	0.004^∗∗^
SD/D	0.09	T_Angio	0.09	0.019^∗^
WF_Warm	0.08	WF_Warm	0.08	0.024^∗^
Biovol	0.07	pH	0.07	0.031^∗^
Cond	0.06	Cond	0.06	0.026^∗^
WF_Spring	0.06	LCFR	0.04	0.095
D	0.05	T_Charo	0.04	0.161
pH	0.05	WF_Spring	0.04	0.171
WF_Cold	0.05	SD/D	0.04	0.243
T_Charo	0.04	WF_Cold	0.03	0.237
TSB	0.03	TP	0.02	0.317
LCFR	0.03	D	0.02	0.663
DIN	0.02	DIN	0.01	0.605
TP	0.02	TSB	0.01	0.810

*Asterisks highlight significant variables (^∗^p < 0.05; ^∗∗^p < 0.01). For variable abbreviations, see **Table 1**.*

### Predictors of SAV Pond-Scale Cover

**FIGURE 8:**
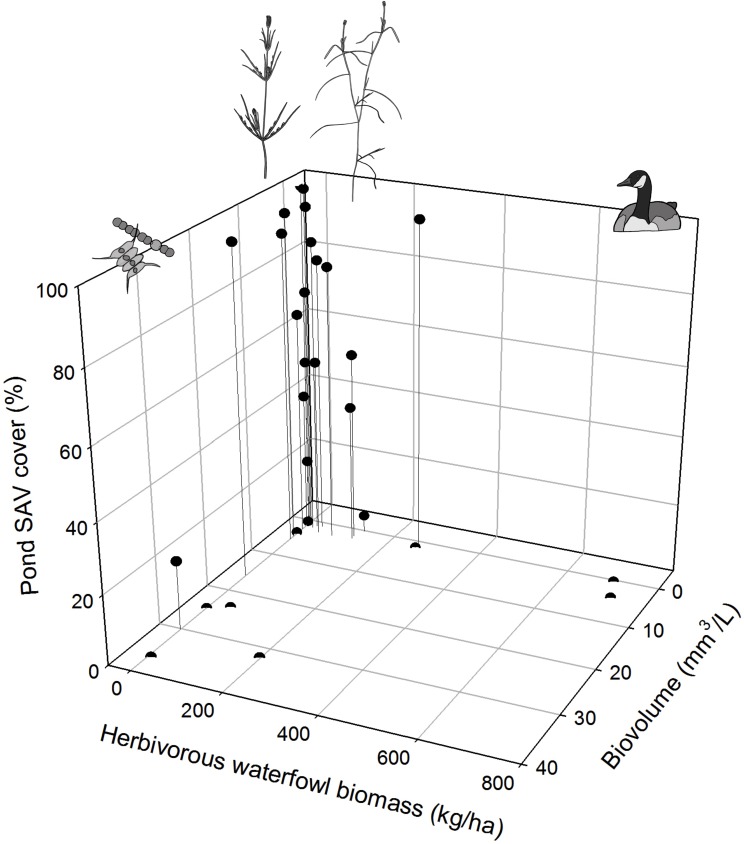
Maximal pond-scale SAV cover in relation to herbivory-related waterfowl density in the warm period (WF_Warm) and phytoplankton biovolume.

We obtained a shortlist of four candidate variables displaying significant singular relationships with SAV cover: phytoplankton biovolume (Biovol), pH, angiosperm propagule density in top layer (T_Angio) and waterfowl herbivory in the warm period (WF_Warm). The maximal summer extent of submerged macrophytes in ponds showed a positive trend with pH (CI range: unidirectionally positive) and a negative relationship with phytoplankton biovolume and waterfowl herbivory in the warm period (CI range: unidirectionally negative; **Table 4** and **Figure 8**). The evidence for an effect of density of angiosperm propagules in the upper sediment layer is less convincing (i.e., CI range bidirectional relative to zero value; **Table 4**).

**Table 4:** Summary of multimodel inference after multiple regression with SAV pond-scale cover as dependent variable (*n* = 29).

Parameter	Averaged estimate	Unconditional SE	Unconditional CI	Relative importance
Intercept	-0.41	0.21	(-0.82, 0.01)	
Biovol	-1.08	0.24	(-1.56, -0.60)^∗^	1.000
pH	0.92	0.24	(0.45, 1.39)^∗^	0.990
T_Angio	0.27	0.23	(-0.18, 0.71)	0.274
WF_Warm	-0.70	0.21	(-1.11, -0.28)^∗^	0.971

*Confidence intervals (CI) marked with ^∗^ indicate a unidirectional relationship with the predictor variable, suggesting a true effect on SAV cover. Relative importance based on summed AICc (Akaike Information Criterion for finite sample size) weights of models containing the variable.*

## Discussion

### Influence of Abiotic Variables and Time Since Biomanipulation

Apart from conductivity and pH, none of the abiotic variables included in the analyses were associated with characteristics of macrophyte composition and abundance. Acidity values were lower in ponds dominated by SAV, especially coinciding with high abundance of tall *Potamogeton pectinatus* (in agreement with Capers et al., 2010) and *P. pusillus*. Nevertheless, pH reached high levels in turbid ponds as well and correlated significantly with oxygen concentrations, indicating decreasing acidity should be interpreted as a by-product of carbon consumption in the process of primary production.

Conductivity was generally relatively low in macrophyte-dominated ponds and high in clear, vegetationless ponds, though not consistently. Possibly, conductivity values were a reflection of a combined effect of photosynthetic activity (Pedersen et al., 2013) and the source of water supply to the waterbody. Although higher values potentially indicate reception of contaminated runoff (Hatt et al., 2004), conductivity remained equivalent to freshwater conditions, and most likely did not profoundly influence macrophyte species filtering or growth. The only sign of a potential structuring effect of accumulated (de-icing or other) salts within the study area was the co-occurrence of high conductivity in WPk1 and proliferation of *Enteromorpha intestinalis*, a euryhaline, yet predominantly brackish-water macroalgal species (Martins et al., 1999; Gallego et al., 2015).

Nutrients presumably were not relevant since phosphorus is the primary limiting element in many freshwater ecosystems (Correll, 1998), yet readily available in the strongly eutrophic ponds within the study area (Peretyatko et al., 2012; Teissier et al., 2012). The elapsed time since TSB (more specifically, water drawdown combined with fish removal) did not affect macrophyte dynamics. Søndergaard et al. (2008) pointed out that successful biomanipulation outcomes in shallow lakes tend to last between 2 and 6 years, followed by gradual deterioration after recovery of fish. The ponds included in our study were mostly biomanipulated within 4 years prior to the analyses, and therefore did not show much relationship with the timing of biomanipulation. With only a partial fish reduction that was executed eight years before the onset of the study, the macrophyte-dominated Tenr pond seemed to be exceptionally resilient to degradation, most likely because of a balanced fish community including a strong piscivorous guild (Leefmilieu Brussel, pers. comm.).

### Influence of Phytoplankton and Zooplankton

As expected, high phytoplankton biovolume was the principal driver explaining submerged macrophyte community composition and pond-scale cover. This is in agreement with relative importance of turbidity in shallow lakes in the Netherlands (Van den Berg et al., 2003; van de Haterd and Ter Heerdt, 2007), and emphasizes the mutually exclusive nature of phytoplankton and submerged macrophyte dominance (Scheffer et al., 1993). In the absence of top-down control of phytoplankton, submerged macrophytes are readily out-competed and will disappear (Scheffer et al., 1993). The results stress the need for continuing management efforts to reduce external and internal loading affecting ponds in the region (Søndergaard et al., 2003; Jeppesen et al., 2005; Hilt et al., 2006).

Although zooplankton plays a paramount role in the control of phytoplankton (Jeppesen et al., 1997, 2005), the estimated filtering rate of large Cladocera (LCFR) did not directly relate to any SAV characteristic. Likely this results from the fact that large *Daphnia* in a number of fishless ponds (Beml and Dens) maintained high water clarity, while SAV did not successfully develop. Similarly, the filtering capacity of zooplankton in vegetated ponds can easily be misjudged if large Cladocera exhibit diel vertical (e.g., to and from *Chara* meadows) or horizontal migration (Burks et al., 2002), or if the contribution of macrophyte-associated cladocerans to total phytoplankton clearance potential is significant (Balayla and Moss, 2004).

### Propagule Banks

Propagule banks in the peri-urban ponds were rarely species-rich and displayed low taxon evenness, because of a dominance of charophycean oospores. Oospores are ubiquitous in many freshwater sediments (de Winton et al., 2000; Bonis and Grillas, 2002; Kalin and Smith, 2007), and high densities might be required for establishment and colonization (Van den Berg et al., 2001). Angiosperm propagules were far less abundant, although any direct comparison with oospore densities would be biased given the small size of oospores. Correcting for propagule dimensions could enable to offset variations in storage capacity, providing a method to evaluate the relative contribution of each species to the propagule bank potential.

Since propagule densities in top- and sub-layers of the sediment were correlated, we focused on the upper 5 cm, supposed to be in close contact with ecological processes in the water column (Dugdale et al., 2001; Bonis and Grillas, 2002; Spencer and Ksander, 2002). However, the extent to which bioturbation by burrowing unionid bivalves, foraging benthivorous fish or waterfowl could mix layers and homogenize strata should not be underestimated (McCall et al., 1995; Vaughn and Hakenkamp, 2001; Huser et al., 2016).

Several elements indicated that the composition and density of the propagule bank plays a significant, albeit modest role in the structuring of SAV in nutrient-enriched ponds. Overall, propagules mainly influenced the assemblage of the macrophyte community (with wealthy stocks of angiosperm propagules accompanying development of small-leaved *Potamogeton* communities), rather than secured SAV dominance.

At the level of individual submerged macrophyte species, the relationship between recruitment reserves and cover within the aquatic vegetation could be summarized as ambiguous. The status of the propagule bank matched maximal vegetation abundance of the most common Angiospermae (*P. pectinatus*, *P. pusillus*, and *Zannichellia palustris*) slightly better than was the case for Charophyceae (indicating some positive influence of high propagule density on vegetation cover), but for each species there were clear dissimilarities depending on the pond. Propagules of *Chara vulgaris* and *Z. palustris* poorly contributed to standing vegetation. Recruitment failure of certain macrophyte species suggests that environmental conditions in the waterbody are not beneficial for germination or growth (the impact of which is mitigated by dormancy of the propagules; Bewley, 1997; Sederias and Colman, 2007; Hay et al., 2008; Nonogaki, 2014).

Local occurrence of submerged macrophytes was not exclusively predicted by presence of propagules, likely because most are capable of vegetative, either non-specialized (e.g., *Ceratophyllum demersum* and *Elodea nuttallii*) or rhizomatous (e.g., *P. pectinatus* and *Z. palustris*), spread and survival (Van Vierssen, 1982; Barrat-Segretain, 1996; Boedeltje et al., 2004; Vári, 2013; Vári and Tóth, 2017). For Charophyceae, establishment without prior availability of oospores would have been less plausible, suggesting that prevailing germination conditions did not always break dormancy (Sederias and Colman, 2007).

Importantly, turbid ponds contained less angiosperm and charophycean propagules in the top sediment layer compared to macrophyte-dominated, clear ponds. Turbid ponds in the Brussels-Capital Region possess high densities of zooplanktivorous and benthivorous fish, resulting in persistent phytoplankton dominance over multiple years and a need for biomanipulation to force a shift. Following a crash of macrophyte domination and winter resetting of autotrophic production, moderate recruitment in spring might start depleting the propagule bank. In case dispersal input does not compensate for the loss, opportunities for submerged macrophyte establishment finally will be lost, leaving the ruined propagule bank as a critical bottleneck for ecosystem recovery. On the contrary, in the shallow Lake Terra Nova (Netherlands), van de Haterd and Ter Heerdt (2007) did not identify propagule limitation after a long period of turbidity. Here, (within-lake) dispersal or less intense phytoplankton blooming could have allowed sparse macrophyte reproduction under turbid conditions. Alternatively, Lake Terra Nova might experience less intense organic matter accumulation on top of propagule-rich sediment when compared to the sapropelium-rich turbid ponds in our study.

Interestingly, propagule densities in clear but sparsely vegetated ponds typically seemed sufficient, though not fully translated as a result of waterfowl herbivory (Beml and Dens in 2009), lemnid cover (VKn1 in 2010), or some other factor affecting SAV. This results in significantly lower similarity between sediment content and vegetation composition when compared to turbid ponds, where submerged macrophytes remain underrepresented in both compartments.

In terms of overall ecological status, the emerging trend seems to be that: (1) for clear, macrophyte-dominated ponds the propagule potential is realized; (2) clear ponds with low SAV cover are unable to utilize their potential; and (3) in turbid ponds recruitment opportunities are inadequate. This is different from patterns observed in interconnected fishing ponds in France, where Arthaud et al. (2012) found no relationship between density of propagules and Chl *a* concentrations in the water, supposedly because propagule banks in the French pond system are regularly replenished – either through dispersal or during recurrent clear-water phases. If propagule banks are known to be impoverished, reproductive success and natural dispersal functioning could be assessed before proceeding to active assistance of macrophyte establishment (Smart et al., 1998; Hilt et al., 2006; Van Onsem et al., 2018).

### Waterfowl Herbivory

During the warm period of the growth season, peaks of plant-consuming waterfowl coincided with negligible SAV abundance, even in the presence of high propagule densities and in the absence of fish or turbidity stress. This confirms the need to incorporate macrophyte herbivory in aquatic research (Bakker et al., 2016; van Altena et al., 2016; Wood et al., 2017). The ranking of waterbird herbivory as second most important biotic predictor following phytoplankton-induced turbidity is consistent with van de Haterd and Ter Heerdt (2007).

It is difficult to situate the avifauna biomass densities obtained for Brussels ponds with respect to other regions, because of the use of peak densities instead of the periodic averages provided by Wood et al. (2012). Nonetheless, maximum densities found in our study seem considerable at times, potentially provoking loss of macrophytes on short notice. The swarming of swans, geese, shelducks, and ducks becomes especially problematic when population sizes overstretch the carrying capacity of the waterbody. In urbanized areas, the pond perimeter can offer a copious supply of staple or alternative food in the form of park lawns and thrown-away bread, thereby attracting and potentially bonding birds to a particular location. Near small, shallow ponds, such an aggregation of opportunistic herbivores lowers the likelihood of large-scale and sustainable SAV establishment.

Nevertheless, the impact of waterfowl seemed to be restricted to direct grazing or disturbance in summer. Although autumn foraging on seeds and tubers or spring grazing on freshly emerged shoots could have had a time-lagged effect (Marklund et al., 2002; Hidding et al., 2010b), high waterfowl biomass density during the spring or the cold season did not weigh on macrophyte performance at the height of the growth season. With an average depth of 0.90 m, the studied ponds are rarely sufficiently deep to prevent herbivory on belowground or freshly emerging biomass. Presumably, waterfowl occurring in the area of ponds was less inclined to forage directly on the water during colder periods. Limited disturbance by waterfowl during the spring establishment phase is in accordance with coot effects found in British broads (Perrow et al., 1997) and a Turkish lake (Sandsten et al., 2005).

Overall, the results illustrate that ecological pond management in densely populated regions should comprise some form of waterfowl control, especially during summer. This can be achieved by prohibiting bird feeding, in combination with regulation of resident, invasive alien waterfowl (specifically *A. aegyptiacus* and *B. canadensis*).

## Conclusion

Given the unambiguous ecological services provided by submerged macrophytes, it is important to understand the relative role of various factors influencing their composition and abundance. We demonstrate that SAV structure depended on three biotic drivers that were tested, namely phytoplankton-induced turbidity, summer waterfowl and propagule banks – in that order of importance. Both turbidity and high summer abundance of large-sized Anatidae species negatively affected submerged macrophyte occurrence. No time-lagged effects of high waterfowl biomass density in cold or spring periods were observed. Propagule banks, on the other hand, displayed poor potential in turbid ponds, indicating an obstacle for establishment. In the studied pond system, rich propagule potential did not guarantee successful colonization, though represented a typical trait of macrophyte-dominated waterbodies and seemed to stimulate small-leaved *Potamogeton* communities. Overall, waterfowl and macrophyte propagule banks are relevant ingredients of pond ecosystem functioning, even within peri-urban areas.

## Author Contributions

The study was conceived and designed by LT and SVO. SVO collected and processed samples, performed statistical analyses and redacted a draft paper, guided, finalized and approved by LT.

## Conflict of Interest Statement

The authors declare that the research was conducted in the absence of any commercial or financial relationships that could be construed as a potential conflict of interest.

## Footnotes

1 bru.observations.be
