# Supplementary material for: Turbidity, Waterfowl Herbivory, and Propagule Banks Shape Submerged Aquatic Vegetation in Ponds
Source: Front Plant Sci. 2018 Oct 16;9:1514. doi: 10.3389/fpls.2018.01514 (PMC6232765; doi:10.3389/fpls.2018.01514)
Supplement: Supplementary file 1 [file Data_Sheet_1.docx]

Supplementary Material

Turbidity, waterfowl herbivory, and propagule banks shape submerged aquatic vegetation in ponds

Stijn Van Onsem*, Ludwig Triest

*** Correspondence: Stijn Van Onsem**: svonsem@gmail.com

The following pages provide information on biotic and abiotic pond characteristics during two consecutive years. In addition, waterfowl counts are listed, as well as ordination summary results.

Measurements of abiotic and biotic variables in 16 ponds in 2009. Ecological status, based on SAV cover and phytoplankton biovolume, is indicated: CH – Clear pond with high SAV cover; CL – Clear pond with low SAV cover; TU – Turbid ponds. See Table 1 for variable abbreviations. N/A – Not available because of complete drawdown for biomanipulation.

| Type | Variable abbrevation | Unit | 2009 | Beml | Dens | Leyb-a | Leyb-b | MlGr | MlKl | PchR | PRB1 | PRB2 | Prm2 | Sbsk | Tenr | VKn1 | VKn2 | WPk1 | Wtml |
| --- | --- | --- | --- | --- | --- | --- | --- | --- | --- | --- | --- | --- | --- | --- | --- | --- | --- | --- | --- |
|  |  |  |  |  |  |  |  |  |  |  |  |  |  |  |  |  |  |  |  |
|  |  |  |  |  |  |  |  |  |  |  |  |  |  |  |  |  |  |  |  |
| Abiotic |  |  |  |  |  |  |  |  |  |  |  |  |  |  |  |  |  |  |  |
| Physical-chemical | Cond | μS/cm |  | 815 | 387 | 547 | 589 | N/A | N/A | 636 | 939 | 748 | N/A | 566 | 463 | 430 | 448 | 942 | 408 |
|  | O_2_ | mg O_2_/L |  | 3.8 | 8.5 | 10.8 | 12.7 | N/A | N/A | 10.5 | 13.0 | 9.2 | N/A | 9.3 | 12.1 | 7.1 | 11.0 | 9.2 | 12.5 |
|  | pH |  |  | 7.5 | 7.9 | 8.6 | 8.2 | N/A | N/A | 8.2 | 8.7 | 8.2 | N/A | 8.1 | 8.2 | 7.5 | 7.9 | 7.8 | 8.4 |
| Nutrients | DIN | mg N/L |  | 0.824 | 0.309 | 0.073 | 0.119 | N/A | N/A | 0.020 | 0.014 | 0.021 | N/A | 0.052 | 0.187 | 0.127 | 0.220 | 0.281 | 0.216 |
|  | NH_4_^+^ | mg N/L |  | 0.782 | 0.294 | 0.058 | 0.035 | N/A | N/A | 0.019 | 0.011 | 0.019 | N/A | 0.047 | 0.139 | 0.071 | 0.184 | 0.226 | 0.154 |
|  | NO_x_ | mg N/L |  | 0.042 | 0.014 | 0.016 | 0.084 | N/A | N/A | 0.001 | 0.003 | 0.002 | N/A | 0.005 | 0.048 | 0.056 | 0.036 | 0.056 | 0.062 |
|  | SRP | mg P/L |  | 0.051 | 0.051 | 0.424 | 0.049 | N/A | N/A | 0.030 | 0.256 | 0.019 | N/A | 0.063 | 0.033 | 0.031 | 0.177 | 0.055 | 0.018 |
|  | TP | mg P/L |  | 0.203 | 0.272 | 0.707 | 0.152 | N/A | N/A | 0.350 | 0.444 | 0.365 | N/A | 0.257 | 0.136 | 0.128 | 0.261 | 0.237 | 0.087 |
| Physical | D | m |  | 1.0 | 0.7 | 0.6 | 0.8 | N/A | N/A | 1.0 | 1.3 | 0.6 | N/A | 0.7 | 0.8 | 1.2 | 1.1 | 1.1 | 1.3 |
|  | SD | m |  | 1.5 | 0.6 | 1.6 | 1.8 | N/A | N/A | 0.3 | 1.6 | 0.2 | N/A | 0.9 | 1.4 | 1.0 | 1.2 | 1.2 | 1.2 |
|  | SD/D |  |  | 1.5 | 0.9 | 2.7 | 2.2 | N/A | N/A | 0.3 | 1.2 | 0.4 | N/A | 1.3 | 1.7 | 0.9 | 1.1 | 1.1 | 0.9 |
| Pond characteristics | TSB | years |  | 2 | 2 | 0 | 0 | N/A | N/A | 10 | 0 | 2 | N/A | 2 | 8 | 4 | 2 | 2 | 4 |
|  |  |  |  |  |  |  |  |  |  |  |  |  |  |  |  |  |  |  |  |
|  |  |  |  |  |  |  |  |  |  |  |  |  |  |  |  |  |  |  |  |
| Biotic |  |  |  |  |  |  |  |  |  |  |  |  |  |  |  |  |  |  |  |
| Phytoplankton | Chl *a* + phaeo | μg/L |  | 3.4 | 20.2 | 29.5 | 18.2 | N/A | N/A | 92.1 | 15.8 | 112.9 | N/A | 14.8 | 15.9 | 117.2 | 33.7 | 11.2 | 15.8 |
|  | Biovol | mm^3^/L |  | 0.3 | 4.2 | 4.8 | 2.8 | N/A | N/A | 23.5 | 2.4 | 36.6 | N/A | 1.7 | 2.6 | 5.7 | 0.6 | 1.2 | 1.2 |
| Zooplankton | LCL | mm |  | 1.27 | 1.74 | 0.81 | 1.10 | N/A | N/A | 0.57 | 0.40 | 0.60 | N/A | 0.77 | 0.66 | 0.91 | 0.81 | 1.06 | 0.37 |
|  | LCD | n/L |  | 29.7 | 140.0 | 111.3 | 14.0 | N/A | N/A | 23.3 | 0.3 | 60.1 | N/A | 566.3 | 108.0 | 5.1 | 9.0 | 247.0 | 11.7 |
|  | LCFR | mL/L/day |  | 368.6 | 3382.5 | 1106.9 | 395.6 | N/A | N/A | 26.8 | 0.3 | 51.7 | N/A | 1681.7 | 920.8 | 16.8 | 34.1 | 2339.6 | 86.4 |
| Propagule bank | T_Charo | n/L |  | 276.2 | 922.4 | 1274.3 | 429.3 | N/A | N/A | 0.0 | 1.0 | 1.0 | N/A | 26.8 | 77.1 | 612.9 | 28.9 | 0.0 | 337.6 |
|  | S_Charo | n/L |  | 263.3 | 399.6 | 328.6 | 32.1 | N/A | N/A | 0.0 | 0.0 | 0.5 | N/A | 77.6 | 46.7 | 101.9 | 1.2 | 0.5 | 46.7 |
|  | T_Angio | n/L |  | 0.5 | 4.8 | 28.6 | 25.7 | N/A | N/A | 0.0 | 0.0 | 0.0 | N/A | 29.3 | 21.9 | 6.1 | 0.0 | 0.0 | 84.3 |
|  | S_Angio | n/L |  | 0.5 | 2.4 | 12.9 | 10.7 | N/A | N/A | 0.0 | 0.0 | 0.0 | N/A | 19.5 | 2.8 | 0.0 | 0.0 | 0.5 | 37.1 |
|  | T_Shannon | H' |  | 0.01 | 0.34 | 0.40 | 0.76 | N/A | N/A | 0.00 | 0.00 | 0.00 | N/A | 0.51 | 0.73 | 0.11 | 0.20 | 0.00 | 1.07 |
|  | S_Shannon | H' |  | 0.04 | 0.42 | 0.85 | 0.77 | N/A | N/A | 0.00 | 0.00 | 0.00 | N/A | 0.55 | 0.49 | 0.43 | 0.00 | 0.00 | 1.03 |
| Macrophytes | MF_FM | % |  | 12.0 | 0.0 | 0.0 | 0.0 | N/A | N/A | 0.0 | 0.0 | 0.0 | N/A | 46.2 | 0.0 | 136.4 | 0.1 | 10.9 | 19.6 |
|  | SAV max | % |  | 0.0 | 0.0 | 95.0 | 80.0 | N/A | N/A | 0.0 | 40.0 | 0.0 | N/A | 95.0 | 65.0 | 90.0 | 99.0 | 20.0 | 85.0 |
| Waterfowl | WF_Cold | kg/ha |  | 252.9 | 0.5 | 0.0 | 0.0 | N/A | N/A | 0.0 | 159.7 | 159.5 | N/A | 644.0 | 27.9 | 18.8 | 0.0 | 21.3 | 36.3 |
|  | WF_Spring | kg/ha |  | 98.7 | 9.3 | 34.9 | 3.7 | N/A | N/A | 0.0 | 173.4 | 96.7 | N/A | 3.2 | 44.8 | 6.7 | 1.3 | 0.4 | 12.3 |
|  | WF_Warm | kg/ha |  | 703.3 | 722.9 | 0.0 | 73.2 | N/A | N/A | 41.8 | 119.1 | 6.6 | N/A | 16.7 | 0.4 | 0.9 | 1.3 | 1.7 | 22.6 |
|  |  |  |  |  |  |  |  |  |  |  |  |  |  |  |  |  |  |  |  |
|  |  |  |  |  |  |  |  |  |  |  |  |  |  |  |  |  |  |  |  |
| Ecological status |  |  |  | CL | CL | CH | CH | N/A | N/A | TU | CH | TU | N/A | CH | CH | CH | CH | CL | CH |
|  |  |  |  |  |  |  |  |  |  |  |  |  |  |  |  |  |  |  |  |

Measurements of abiotic and biotic variables in 16 ponds in 2010. Ecological status, based on SAV cover and phytoplankton biovolume, is indicated: CH – Clear pond with high SAV cover; CL – Clear pond with low SAV cover; TU – Turbid ponds. See Table 1 for variable abbreviations.

| Type | Variable abbrevation | Unit | 2010 | Beml | Dens | Leyb-a | Leyb-b | MlGr | MlKl | PchR | PRB1 | PRB2 | Prm2 | Sbsk | Tenr | VKn1 | VKn2 | WPk1 | Wtml |
| --- | --- | --- | --- | --- | --- | --- | --- | --- | --- | --- | --- | --- | --- | --- | --- | --- | --- | --- | --- |
|  |  |  |  |  |  |  |  |  |  |  |  |  |  |  |  |  |  |  |  |
|  |  |  |  |  |  |  |  |  |  |  |  |  |  |  |  |  |  |  |  |
| Abiotic |  |  |  |  |  |  |  |  |  |  |  |  |  |  |  |  |  |  |  |
| Physical-chemical | Cond | μS/cm |  | 862 | 466 | 572 | 584 | 630 | 497 | 631 | 818 | 737 | 896 | 750 | 454 | 496 | 374 | 923 | 481 |
|  | O_2_ | mg O_2_/L |  | 4.0 | 6.1 | 8.6 | 19.4 | 9.9 | 9.0 | 11.8 | 12.3 | 10.0 | 6.8 | 5.2 | 14.3 | 5.1 | 16.4 | 9.7 | 11.4 |
|  | pH |  |  | 7.5 | 7.6 | 8.4 | 9.0 | 7.9 | 8.4 | 8.3 | 9.6 | 8.2 | 7.7 | 7.6 | 8.5 | 7.3 | 8.8 | 7.7 | 7.9 |
| Nutrients | DIN | mg N/L |  | 0.732 | 1.241 | 0.022 | 0.031 | 0.267 | 0.047 | 0.022 | 0.043 | 0.021 | 0.363 | 0.649 | 0.053 | 0.183 | 0.033 | 0.363 | 0.025 |
|  | NH_4_^+^ | mg N/L |  | 0.711 | 1.166 | 0.018 | 0.023 | 0.187 | 0.038 | 0.018 | 0.036 | 0.016 | 0.243 | 0.635 | 0.048 | 0.161 | 0.026 | 0.115 | 0.019 |
|  | NO_x_ | mg N/L |  | 0.021 | 0.075 | 0.003 | 0.008 | 0.079 | 0.009 | 0.004 | 0.007 | 0.005 | 0.120 | 0.014 | 0.005 | 0.022 | 0.007 | 0.248 | 0.005 |
|  | SRP | mg P/L |  | 0.111 | 0.058 | 1.262 | 0.226 | 0.018 | 0.087 | 0.007 | 0.230 | 0.004 | 0.554 | 0.079 | 0.067 | 0.076 | 0.035 | 0.010 | 0.063 |
|  | TP | mg P/L |  | 0.425 | 0.221 | 1.923 | 0.940 | 0.142 | 0.243 | 0.316 | 0.376 | 0.240 | 1.041 | 0.282 | 0.267 | 0.278 | 0.130 | 0.107 | 0.208 |
| Physical | D | m |  | 1.0 | 0.7 | 0.6 | 0.8 | 0.9 | 0.9 | 0.9 | 1.1 | 0.6 | 0.6 | 0.5 | 0.8 | 1.1 | 1.1 | 1.0 | 1.1 |
|  | SD | m |  | 1.5 | 1.7 | 1.1 | 0.3 | 1.0 | 1.4 | 0.3 | 1.9 | 0.2 | 1.6 | 1.5 | 1.2 | 1.4 | 1.6 | 0.9 | 1.6 |
|  | SD/D |  |  | 1.4 | 2.5 | 1.9 | 0.4 | 1.1 | 1.7 | 0.3 | 1.6 | 0.4 | 2.6 | 3.2 | 1.7 | 1.4 | 1.5 | 0.9 | 1.5 |
| Pond characteristics | TSB | years |  | 3 | 3 | 1 | 1 | 1 | 1 | 10 | 1 | 3 | 1 | 3 | 9 | 5 | 3 | 3 | 5 |
|  |  |  |  |  |  |  |  |  |  |  |  |  |  |  |  |  |  |  |  |
|  |  |  |  |  |  |  |  |  |  |  |  |  |  |  |  |  |  |  |  |
| Biotic |  |  |  |  |  |  |  |  |  |  |  |  |  |  |  |  |  |  |  |
| Phytoplankton | Chl *a* + phaeo | μg/L |  | 3.1 | 20.5 | 108.7 | 257.0 | 13.0 | 6.3 | 96.7 | 8.5 | 217.7 | 48.7 | 11.6 | 15.9 | 35.5 | 12.4 | 24.4 | 6.5 |
|  | Biovol | mm^3^/L |  | 0.2 | 1.6 | 16.2 | 30.2 | 1.8 | 0.7 | 24.7 | 0.8 | 32.1 | 4.0 | 1.5 | 2.6 | 1.2 | 1.0 | 1.3 | 1.1 |
| Zooplankton | LCL | mm |  | 1.07 | 1.51 | 0.00 | 0.20 | 0.97 | 1.06 | 0.39 | 0.44 | 0.61 | 0.92 | 1.51 | 0.66 | 0.94 | 0.50 | 1.27 | 1.42 |
|  | LCD | n/L |  | 143.3 | 52.7 | 0.0 | 0.1 | 70.3 | 25.2 | 5.2 | 0.1 | 5.1 | 1.7 | 129.1 | 108.0 | 27.8 | 8.8 | 20.0 | 5.8 |
|  | LCFR | mL/L/day |  | 1155.4 | 1032.0 | 0.0 | 0.1 | 466.9 | 183.1 | 7.1 | 0.2 | 9.1 | 6.1 | 3854.5 | 920.8 | 458.3 | 27.7 | 355.5 | 19.7 |
| Propagule bank | T_Charo | n/L |  | 276.2 | 922.4 | 1274.3 | 429.3 | 448.9 | 0.0 | 0.0 | 1.0 | 1.0 | 0.5 | 26.8 | 77.1 | 612.9 | 28.9 | 0.0 | 337.6 |
|  | S_Charo | n/L |  | 263.3 | 399.6 | 328.6 | 32.1 | 525.7 | 0.5 | 0.0 | 0.0 | 0.5 | 0.5 | 77.6 | 46.7 | 101.9 | 1.2 | 0.5 | 46.7 |
|  | T_Angio | n/L |  | 0.5 | 4.8 | 28.6 | 25.7 | 50.0 | 5.2 | 0.0 | 0.0 | 0.0 | 0.0 | 29.3 | 21.9 | 6.1 | 0.0 | 0.0 | 84.3 |
|  | S_Angio | n/L |  | 0.5 | 2.4 | 12.9 | 10.7 | 48.1 | 9.5 | 0.0 | 0.0 | 0.0 | 0.0 | 19.5 | 2.8 | 0.0 | 0.0 | 0.5 | 37.1 |
|  | T_Shannon | H' |  | 0.01 | 0.34 | 0.40 | 0.76 | 0.62 | 0.00 | 0.00 | 0.00 | 0.00 | 0.00 | 0.51 | 0.73 | 0.11 | 0.20 | 0.00 | 1.07 |
|  | S_Shannon | H' |  | 0.04 | 0.42 | 0.85 | 0.77 | 0.61 | 0.23 | 0.00 | 0.00 | 0.00 | 0.00 | 0.55 | 0.49 | 0.43 | 0.00 | 0.00 | 1.03 |
| Macrophytes | MF_FM | % |  | 25.3 | 0.0 | 0.0 | 0.0 | 0.0 | 6.8 | 0.0 | 4.9 | 0.0 | 23.3 | 62.3 | 0.6 | 175.0 | 3.3 | 38.7 | 7.6 |
|  | SAV max | % |  | 5.0 | 0.0 | 95.0 | 20.0 | 55.0 | 70.0 | 0.0 | 95.0 | 0.0 | 0.5 | 40.0 | 51.9 | 1.0 | 100.0 | 50.0 | 80.0 |
| Waterfowl | WF_Cold | kg/ha |  | 616.0 | 190.2 | 300.4 | 68.5 | 247.9 | 63.2 | 70.3 | 96.1 | 219.6 | 35.0 | 568.9 | 64.9 | 16.0 | 1.9 | 27.8 | 49.1 |
|  | WF_Spring | kg/ha |  | 0.0 | 178.0 | 0.0 | 6.6 | 67.5 | 49.8 | 24.5 | 0.0 | 36.6 | 0.0 | 0.0 | 28.2 | 9.7 | 0.0 | 109.4 | 10.3 |
|  | WF_Warm | kg/ha |  | 128.1 | 262.0 | 0.0 | 0.0 | 118.0 | 0.0 | 0.0 | 262.4 | 197.3 | 0.0 | 0.0 | 39.8 | 0.0 | 0.0 | 2.5 | 33.5 |
|  |  |  |  |  |  |  |  |  |  |  |  |  |  |  |  |  |  |  |  |
| Ecological status |  |  |  | CL | CL | CH | TU | CH | CH | TU | CH | TU | CL | CH | CH | CL | CH | CH | CH |
|  |  |  |  |  |  |  |  |  |  |  |  |  |  |  |  |  |  |  |  |

Abundance of herbivorous waterfowl species in 16 ponds in the period 2008-2010. Values represent the maximal simultaneously observed bird numbers within a given period.


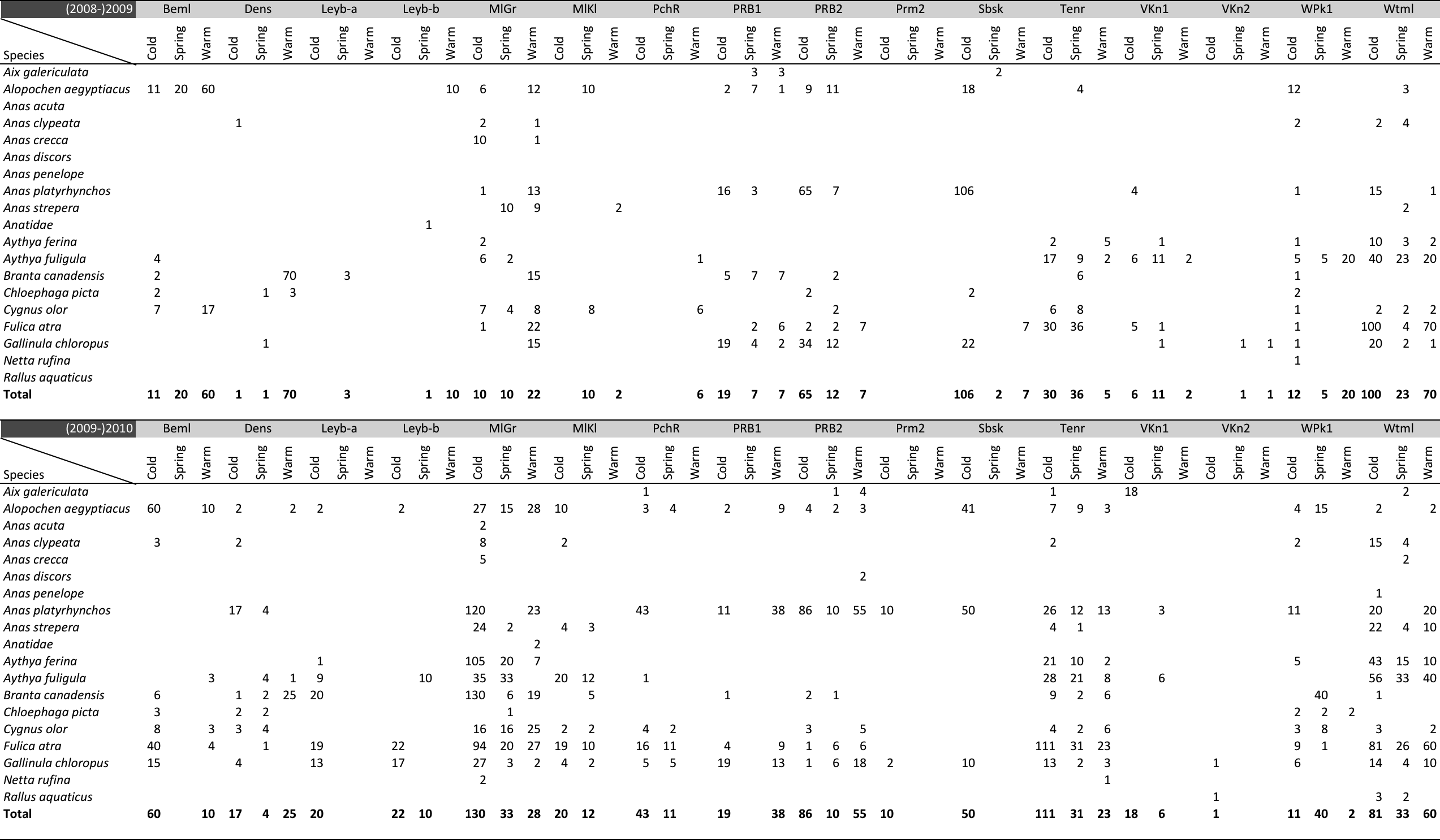


RDA summary results for first four axes of the ordination of macrophyte communities in function of environmental variables.

| Axes | 1 | 2 | 3 | 4 | Total variance |
| --- | --- | --- | --- | --- | --- |
|  |  |  |  |  |  |
| Eigenvalues | 0.243 | 0.135 | 0.067 | 0.061 | 1 |
| Species-environment correlations | 0.858 | 0.924 | 0.768 | 0.867 |  |
| Cumulative percentage variance |  |  |  |  |  |
| of species data | 24.3 | 37.8 | 44.5 | 50.6 |  |
| of species-environment relation | 37.3 | 58.1 | 68.4 | 77.7 |  |
|  |  |  |  |  |  |
| Sum of all eigenvalues |  |  |  |  | 1 |
| Sum of all canonical eigenvalues |  |  |  |  | 0.651 |
